# The global disease burden attributable to unsafe water, sanitation, and handwashing with unqualified facilities from 1990 to 2019

**DOI:** 10.7189/jogh.14.04162

**Published:** 2024-08-23

**Authors:** Hui Zeng, Hui Gan, Yuanru Liu, Baoqing Sun

**Affiliations:** 1 Department of Clinical Laboratory of the First Affiliated Hospital of Guangzhou Medical University, State Key Laboratory of Respiratory Disease, National Center for Respiratory Medicine, National Clinical Research Center for Respiratory Disease, Guangzhou Institute of Respiratory Health, Guangzhou, China; 2 Guangzhou Medical University, Guangzhou, China

## Abstract

**Background:**

Water, sanitation, and hygiene (WaSH) are crucial determinants of human health. However, the spatio-temporal trends in the global burden of disease attributable to unsafe WaSH remain poorly understood. This study aimed to estimate the disease burden attributable to unsafe WaSH from 1990 to 2019 using data from the Global Burden of Disease (GBD) Study 2019, providing new insights into the associated health conditions.

**Methods:**

We extracted data on deaths and disability-adjusted life years (DALYs) attributable to unsafe WaSH from 1990 to 2019 from the GBD 2019. The disease burden was evaluated by region, sociodemographic index (SDI), sex, age, risk factor, and specific disease.

**Results:**

Globally, unsafe WaSH was responsible for 1 656 887.37 (95% uncertainty interval (UI) = 1 198 864.94, 2 312 688.33) deaths in 2019, a 49% decrease from 1990. The global age-standardised DALY rate due to unsafe WaSH was 1244.29 (95% UI = 993.20, 1544.13) per 100 000 in 2019, a 66% reduction since 1990. Western sub-Saharan Africa had the highest age-standardised death rate (ASDR) and age-standardised DALY rate in both 1990 and 2019. Among the 21 regions studied, only high-income North America witnessed an increasing ASDR from 1990 to 2019. Countries and territories in low SDI regions had higher ASDRs and age-standardised DALY rates. U-shaped associations were observed between the estimated annual percentage change (EAPC) of ASDR, EAPC of age-standardised DALY rate, and SDI. Both rates were slightly lower in females, with the burden concentrated in those under five and over 80 years old. In 2019, unsafe water source and diarrhoeal diseases remained the leading risk factor and cause of unsafe WaSH-related disease burden, respectively.

**Conclusions:**

Despite substantial improvements in hygiene awareness and health education, unsafe WaSH persists as a significant global health risk and a major contributor to the burden of diarrhoeal diseases. Disparities across regions and age groups remain evident. Increased efforts are needed to raise awareness and strengthen water and sanitation infrastructure, particularly in low SDI settings, to mitigate the health risks associated with unsafe WaSH.

Water, sanitation, and hygiene (WaSH) constitute a critical global environmental issue [1]. In an effort to substantially improve global access to safe water and sanitation, the United Nations (UN) General Assembly, with 193 member states, adopted Sustainable Development Goal (SDG) six targeting drinking water, sanitation, and hygiene [2]. Sustainable Development Goal 6 encompasses two specific targets: (a) Target 6.1: by 2030, achieve universal and equitable access to safe and affordable drinking water for all; (b) Target 6.2: by 2030, achieve access to adequate and equitable sanitation and hygiene for all, end open defecation, and pay special attention to the needs of women, girls, and those in vulnerable situations.

Despite the implementation of WaSH measures in households and public settings, such as schools and health care facilities, and the emphasis on sanitation and hygiene to prevent disease transmission during the coronavirus disease 2019 pandemic [3], some regions continue to face challenges in accessing these services. Factors such as poverty, inequality, lack of basic water purification facilities, and limited health education contribute to these ongoing struggles [4]. Unsafe WaSH conditions have been well-documented to be associated with premature death and various diseases, including an increased risk of diarrhoea [5], lower respiratory infections [6], malaria [7], and soil-transmitted helminthiases [8]. Moreover, compromised WaSH conditions can also adversely impact economic outcomes [9], educational attainment, cognitive development, and overall well-being, including mental health [10,11]. Wolf et al. [12] estimated that providing universal access to safe WaSH could prevent approximately 1.4 million deaths and 74 million disability-adjusted life years (DALYs), accounting for 2.5% of all global deaths and 2.9% of all DALYs. Notably, children under five years of age bore a disproportionate burden, representing 7.6% of all deaths and 7.5% of all DALYs attributable to unsafe WaSH.

In this study, we investigated the global burden of disease attributable to unsafe WaSH using data on deaths and DALYs from the Global Burden of Diseases, Injuries, and Risk Factors (GBD) Study 2019, spanning from 1990 to 2019. Our analysis provides new insights into the distribution of this burden across regions, sociodemographic index (SDI) levels, sexes, and age groups, while also highlighting some under-explored areas within the field of WaSH research.

## METHODS

### Overview

The Global Burden of Disease (GBD) Study 2019, conducted by the Institute for Health Metrics and Evaluation (IHME), has represented the most comprehensive and rigorous effort to quantify global health trends and disease burdens to date. The study, which adheres to the Guidelines for Accurate and Transparent Health Estimates Reporting (GATHER) statement, analyses 369 diseases and injuries, 286 causes of death, 364 non-fatal health conditions, and 87 risk factors across 204 countries and territories [13,14]. The GBD Study utilises data from the Global Health Data Exchange (GHDx) (http://ghdx.healthdata.org/gbd-results-tool), which includes census records, scientific literature, environmental monitoring, and disease registries. In contrast, the World Health Organization (WHO) primarily relies on real-time data updates from national health institutions of member states and the Centers for Disease Control and Prevention (CDC). Although the WHO has conducted studies on WaSH, the GBD database offers a more comprehensive and diverse data source for estimating global health burdens.

### Data sources

Publicly available data on the global burden of disease attributable to unsafe WaSH were obtained from the Global Health Data Exchange (GHDx) (http://ghdx.healthdata.org/gbd-results-tool), an online data repository, following the operational guidelines provided by the GBD Study. We extracted data on the number of deaths and DALYs, as well as their age-standardised rates and corresponding 95% uncertainty intervals (UIs), for unsafe WaSH. Detailed information on the data collection process can be found at https://www.healthdata.org/data-tools-practices/data-collection.

### Measures of burden

The burden of disease attributable to unsafe WaSH was quantified using deaths, disability-adjusted life years (DALYs), and their respective age-standardised rates at the global, regional, and national levels. Disability-adjusted life years, a composite metric combining years of life lost (YLLs) and years lived with disability (YLDs), represent the total number of healthy life years lost from the onset of disease until death. Years of life lost measure the number of years lost due to premature mortality caused by unsafe WaSH-related diseases, while YLDs account for the duration and severity of non-fatal health outcomes associated with unsafe WaSH. Age-standardised death rates (ASDRs) and age-standardised DALY rates were employed to ensure comparability across populations with varying age structures. These rates were calculated by dividing the number of deaths or DALYs in each age group attributable to unsafe WaSH by the corresponding age-specific population, multiplying the result by 100 000, and then summing these values after multiplying them by the standard population weights for each age group. Temporal trends in ASDRs and age-standardised DALY rates were assessed using the estimated annual percentage change (EAPC) [15]. Estimated annual percentage change was derived from the slope (β) of a linear regression model, y = α + βx + ε, where y = ln(ASR), x = calendar year, and ε = error term, such that EAPC = 100 × (eβ - 1). An EAPC>0 indicated an increasing trend over time, while an EAPC<0 suggested a decreasing trend. An EAPC with a 95% UI containing 0 was considered to indicate no significant change during the study period.

### Data analysis

The burden of death attributable to unsafe WaSH was estimated using the Cause of Death Ensemble model (CODEm) and spatiotemporal Gaussian process regression. Age-standardised death rates (ASDRs) and age-standardised DALY rates from all sources were generated using the Bayesian meta-regression tool DisMod-MR 2.1, ensuring consistent estimates across the data set [13]. Throughout the analysis, data verification and double-checking procedures were implemented to maintain the accuracy of the results. Age-standardised death rates and age-standardised DALY rates were reported as mean values with their corresponding 95% UIs, defined as the 2.5th and 97.5th percentiles of the posterior distribution, consistent with previous studies [16,17]. Pearson correlation coefficient (R) was used to assess the associations among ASDRs, age-standardised DALY rates, and their respective estimated annual percentage changes (EAPCs) in countries with SDI in 2019. *P*-value <0.05 were considered statistically significant. Data analysis and visualisation were performed using Microsoft Office Excel 2022 and GraphPad Prism version 9.1.1 (GraphPad Software, San Diego, CA, USA).

## RESULTS

### Global burden of disease attributable to unsafe WaSH

This section presents the health impact of unsafe WaSH at the global, regional, and national levels. In 2019, the global mortality rate attributable to unsafe WaSH was 22.69 (95% UI = 16.61, 31.24) deaths per 100 000 population, representing a 65% (95% UI = 53.91, 71.67) decline from 63.93 (95% UI = 48.88, 80.82) deaths per 100 000 population in 1990 (**Table 1**). Unsafe WaSH was responsible for approximately 1 656 887.37 (95% UI = 1 198 864.94, 2 312 688.33) deaths worldwide in 2019, a 49% decrease since 1990 (Table S1 in the **Online Supplementary Document**). The global age-standardised DALY rate due to unsafe WaSH decreased by 66%, from 3613.97 (95% UI = 2942.83, 4297.76) per 100 000 in 1990 to 1244.29 (95% UI = 993.20, 1544.13) per 100 000 in 2019 (**Table 1**).

**Table 1:** ASDR and age-standardised DALY rate in 1990 and 2019, and their changes between 1990 and 2019 due to unsafe water, sanitation, and hygiene in different regions and globally

Region	ASDR (per 100 000), No. (95% UI)			Age-standardised DALY rate (per 100 000), No. (95% UI)		
	**1990**	**2019**	**EAPC (%)**	**1990**	**2019**	**EAPC (%)**
Global	63.93 (48.88, 80.82)	22.69 (16.61, 31.24)	−0.65 (−0.72, −0.54)	3613.97 (2942.83, 4297.76)	1244.29 (993.20, 1544.13)	−0.66 (−0.72, −0.57)
Andean Latin America	37.79 (27.64, 49.07)	7.54 (4.52, 11.19)	−0.80 (−0.86, −0.73)	2235.86 (1763.56, 2728.41)	373.96 (268.58, 496.39)	−0.83 (−0.87, −0.79)
Australasia	0.40 (0.20, 0.60)	0.30 (0.17, 0.44)	−0.24 (−0.38, 0.01)	23.95 (14.93, 35.42)	17.67 (9.85, 29.20)	−0.26 (−0.44, −0.06)
Caribbean	44.82 (35.24, 55.04)	16.82 (11.06, 23.73)	−0.62 (−0.73, −0.48)	3213.59 (2602.25, 3918.24)	1245.65 (807.85, 1820.39)	−0.61 (−0.73, −0.45)
Central Asia	19.14 (15.48, 23.11)	2.71 (1.73, 3.85)	−0.86 (−0.89, −0.82)	1680.71 (1374.42, 2018.04)	290.27 (211.75, 380.85)	−0.83 (−0.86, −0.78)
Central Europe	1.29 (0.91, 1.70)	0.34 (0.21, 0.49)	−0.74 (−0.79, −0.68)	203.36 (146.69, 267.84)	68.09 (37.57, 105.47)	−0.67 (−0.75, −0.58)
Central Latin America	33.53 (28.59, 38.02)	5.23 (3.75, 6.72)	−0.84 (−0.88, −0.81)	1934.40 (1654.95, 2204.96)	283.45 (211.27, 357.76)	−0.85 (−0.88, −0.82)
Central sub-Saharan Africa	200.39 (139.78, 278.44)	86.96 (59.42, 128.06)	−0.57 (−0.68, −0.40),	8956.35 (6197.61, 12 129.00)	3293.20 (2258.37, 4601.31)	−0.63 (−0.73, −0.51)
East Asia	14.12 (10.38, 18.23)	0.88 (0.53, 1.28)	−0.94 (−0.95, −0.92)	1001.16 (773.34, 1250.20)	92.42 (65.07, 123.33)	−0.91 (−0.93, −0.87)
Eastern Europe	1.43 (1.07, 1.80)	0.52 (0.28, 0.77)	−0.64 (−0.75, −0.56)	216.86 (163.93, 277.83)	123.52 (81.63, 176.64)	−0.43 (−0.53, −0.34)
Eastern sub-Saharan Africa	231.49 (154.92, 320.92)	88.58 (61.49, 121.34)	−0.62 (−0.72, −0.44)	10 184.59 (7733.17, 12 813.57)	3268.97 (2442.89, 4176.34)	−0.68 (−0.76, −0.57)
High-income Asia Pacific	1.32 (0.74, 1.95)	0.58 (0.30, 0.88)	−0.56 (−0.62, −0.50)	33.21 (20.99, 46.24)	13.12 (7.85, 19.45)	−0.60 (−0.67, −0.53)
High-income North America	0.29 (0.14, 0.45)	0.33 (0.22, 0.44)	0.14 (−0.13, 0.74)	25.83 (16.09, 38.67)	14.68 (9.64, 21.24)	−0.43 (−0.58, −0.24)
North Africa and Middle East	28.44 (21.36, 36.78)	6.72 (4.46, 9.04)	−0.76 (−0.81, −0.71),	2126.24 (1634.98, 2733.73)	555.23 (388.06, 742.81)	−0.74 (−0.80, −0.67)
Oceania	100.58 (70.36, 144.35)	55.32 (36.49, 84.46)	−0.45 (−0.57, −0.31)	3466.25 (2620.09, 4438.90)	2083.80 (1502.79, 2796.66)	−0.40 (−0.54, −0.23)
South Asia	241.56 (160.51, 330.52)	66.06 (39.47, 105.89)	−0.73 (−0.80, −0.60)	7382.54 (5580.93, 9327.32)	1814.33 (1281.30, 2562.24)	−0.75 (−0.81, −0.68)
Southeast Asia	75.09 (52.56, 105.91)	18.46 (12.00, 27.23)	−0.75 (−0.81, −0.67)	3315.61 (2462.07, 4286.16)	640.49 (476.41, 815.41)	−0.81 (−0.86, −0.74)
Southern Latin America	5.24 (3.80, 6.65)	1.99 (1.12, 2.85)	−0.62 (−0.71, −0.56)	280.60 (227.57, 333.73)	83.87 (52.68, 115.68)	−0.70 (−0.78, −0.62)
Southern sub-Saharan Africa	84.47 (62.18, 115.69)	46.94 (31.80, 67.60)	−0.44 (−0.52, −0.35)	4078.97 (3204.08, 5158.04)	1838.64 (1366.72, 2456.77)	−0.55 (−0.62, −0.46)
Tropical Latin America	33.80 (27.76, 40.75)	5.22 (3.57, 6.85)	−0.85 (−0.89, −0.81)	2341.21 (1911.41, 2890.42)	280.04 (211.22, 351.04)	−0.88 (−0.91, −0.85)
Western Europe	0.32 (0.17, 0.48)	0.22 (0.12, 0.32)	−0.33 (−0.39, −0.23)	15.55 (9.96, 22.43)	7.60 (4.74, 11.36)	−0.51 (−0.56, −0.46)
Western sub-Saharan Africa	264.03 (183.51, 348.67)	104.01 (76.29, 140.20)	−0.61 (−0.70, −0.46)	13 904.46 (10 078.84, 17 822.28)	5001.61 (3794.57, 6409.28)	−0.64 (−0.74, −0.50)

ASDR – disability-adjusted death rate, DALY – disability-adjusted life year, EAPC – estimated annual percentage change, UI – uncertainty interval

**Figure 1:**
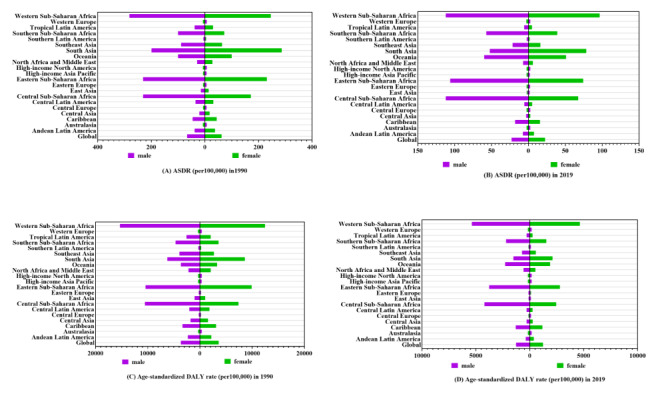
ASDR and age-standardised DALY rate attributable to unsafe water, sanitation, and hygiene with different sexes in different regions and global average levels in 1990 and 2019. Male and female ASDR in different regions and global average levels attributable to unsafe water, sanitation, and hygiene in 1990 (**Panel A**) and in 2019 (**Panel B**). Male and female age-standardised DALY rate in different regions and global average levels attributable to unsafe water, sanitation, and hygiene in 1990 (**Panel C**) and in 2019 (**Panel D**). ASDR – disability-adjusted death rate, DALY – disability-adjusted life year.

Comparing the global average ASDR and age-standardised DALY rate due to unsafe WaSH with those of 21 regions worldwide revealed that, except for high-income North America, which displayed an increase in ASDR in 2019, all other regions exhibited declining trends in both metrics over the study period. The disease burden attributable to unsafe WaSH was heavily concentrated in sub-Saharan Africa, South-East Asia, and South Asia (**Figure 1**). Western sub-Saharan Africa had the highest ASDR (264.03 (95% UI = 183.51, 348.67) per 100 000) and age-standardised DALY rate (13 904.46 (95% UI = 10 078.84, 17 822.28) per 100 000) in 2019 (**Table 1**). No significant differences were observed in the global average mortality and DALY rates between males and females (**Figure 1**).

**Figure 2:**
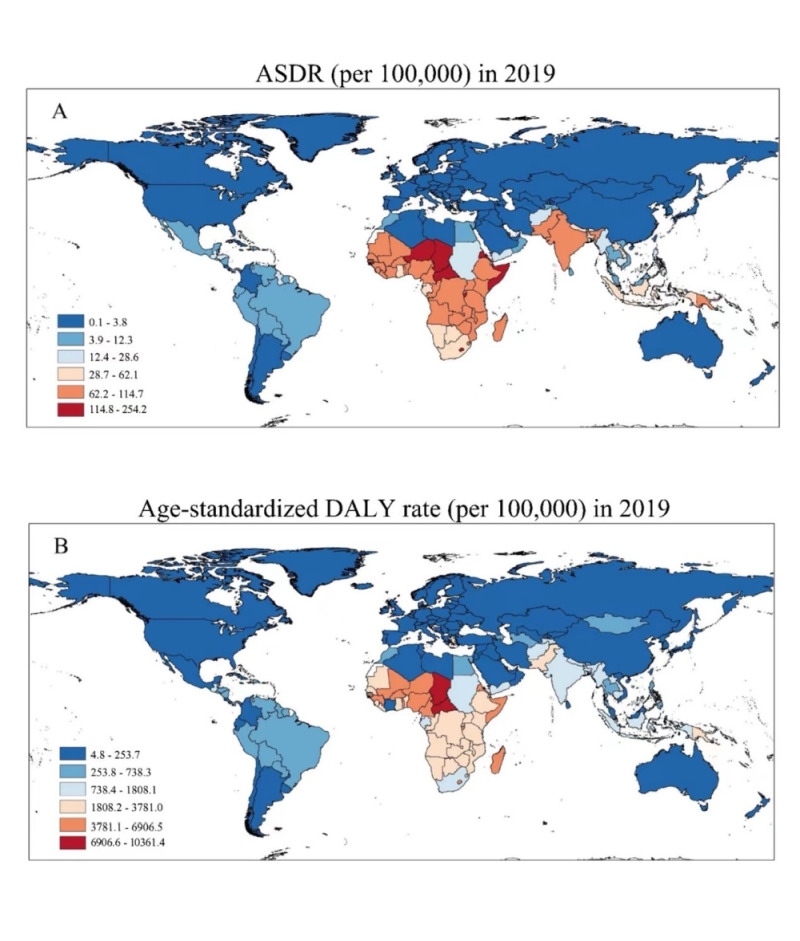
ASDR (**Panel A**) and age-standardised DALY rate (**Panel B**) per 100 000 at national level in 2019 (both sexes, all ages). ASDR – disability-adjusted death rate, DALY – disability-adjusted life year.

At the national level, the highest ASDRs and age-standardised DALY rates in 2019 were observed in Africa and Southeast Asia, particularly in the Central African Republic and Chad (**Figure 2**, panels A–B). The Central African Republic had an ASDR of 254.21 (95% UI = 155.71, 380.43) per 100 000 and an age-standardised DALY rate of 10 361.41 (95% UI = 6769.31, 14 343.01) per 100 000 in 2019, while Chad had an ASDR of 190.27 (95% UI = 136.81, 255.24) per 100 000 and an age-standardised DALY rate of 9817.13 (95% UI = 6944.73, 13 158.07) per 100 000. In contrast, countries such as Finland, San Marino, Italy, Switzerland, and Iceland had considerably lower ASDRs and age-standardised DALY rates compared to the global average **(****Figure 2**, panels A–B; Table S2 in the **Online Supplementary Document**). Over the three decades examined, Niger experienced the largest decrease in age-standardised DALY rate, dropping from 22 407.58 per 100 000 in 1990 to 6906.54 per 100 000 in 2019. However, Equatorial Guinea had the most substantial decline in ASDR, with a 69.77% reduction from 354.33 per 100 000 in 1990 to 107.10 per 100 000 in 2019 (Table S2 in the **Online Supplementary Document**).

### Burden of disease attributable to unsafe WaSH across sociodemographic index (SDI) levels

**Figure 3:**
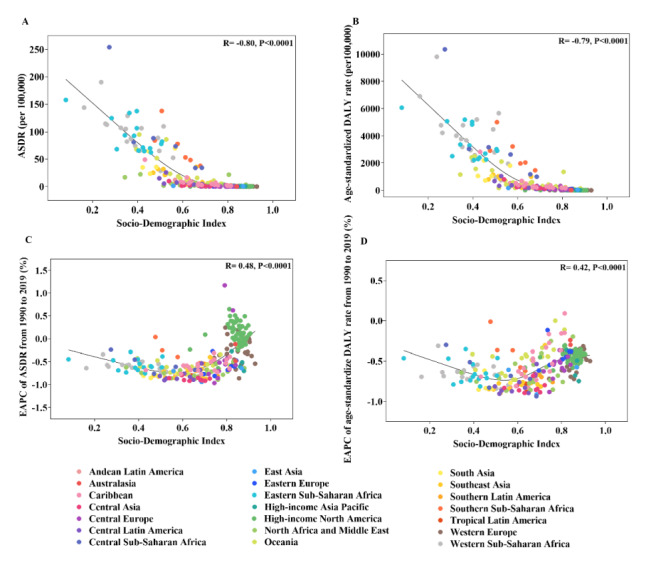
The correlation between ASDR, age-standardised DALY rate, EAPC of ASDR, EAPC of age-standardised DALY rate and SDI of different countries and territories in 21 GBD regions. ASDR (**Panel A**) and age-standardised DALY rate (**Panel B**) in different countries and territories in 21 GBD regions by SDI in 2019. EAPC of ASDR (**Panel C**) and age-standardised DALY rate (**Panel D**) in different countries and territories in 21 GBD regions by SDI from 1990 to 2019. Each dot represented a country or territory and its colour implied the region that the country or territory located. ASDR – disability-adjusted death rate, DALY – disability-adjusted life year, EAPC – estimated annual percentage change, SDI – sociodemographic index.

**Figure 3** presents the age-standardised death rates (ASDRs), age-standardised DALY rates, and their respective estimated annual percentage changes (EAPCs) in countries with different SDI levels in 2019. Both ASDRs and age-standardised DALY rates exhibited strong negative correlations with SDI (R = −0.80, *P* < 0.0001; R = −0.79, *P* < 0.0001, respectively) (**Figure 3**, panels A–B). Conversely, the EAPCs of ASDRs and age-standardised DALY rates showed moderate positive correlations with SDI (R = 0.48, *P* < 0.0001; R = 0.42, *P* < 0.0001, respectively) (**Figure 3**, panels C–D). Notably, U-shaped relationships were observed between the EAPCs of ASDRs, age-standardised DALY rates, and SDI from 1990 to 2019 (**Figure 3**, panels C–D). The lowest EAPCs, indicating the greatest declines in ASDRs and age-standardised DALY rates between 1990 and 2019, were found in countries with an SDI of approximately 0.6. Countries and territories with lower SDI levels tended to have smaller reductions in ASDRs and age-standardised DALY rates compared to those with moderate SDI levels, while higher SDI countries and territories exhibited even smaller reductions or increasing trends.

### Burden of disease attributable to unsafe WaSH by sex and age group

The age-standardised death rates and age-standardised DALY rates attributable to unsafe WaSH decreased in both sexes globally from 1990 to 2019 (Figure S1 in the **Online Supplementary Document**). In 2019, the ASDR was slightly higher in males (22.82 (95% confidence interval (CI) = 17.18, 32.41) per 100 000) than in females (22.60 (95% CI = 14.11, 36.13) per 100 000). Similarly, the age-standardised DALY rate was higher in males (1264.12 (95% CI = 994.28, 1604.83) per 100 000) compared to females (1223.12 (95% CI = 929.09, 1628.82) per 100 000) (Figure S1 in the **Online Supplementary Document**).

**Figure 4:**
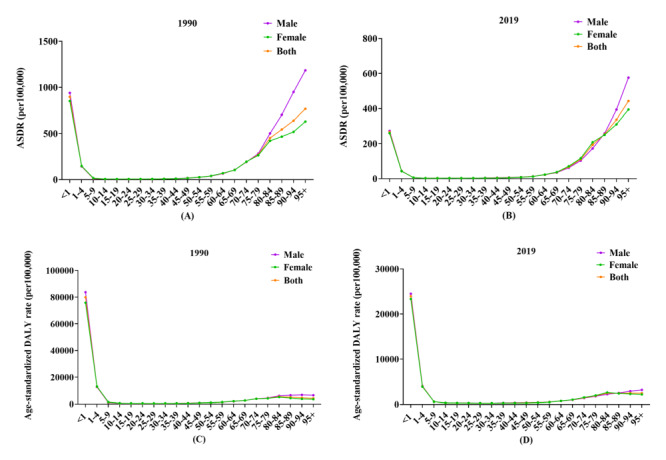
ASDR and age-standardised DALY rate of different age groups due to unsafe water, sanitation, and hygiene in global in 1990 and 2019. ASDR in 1990 (**Panel A**) and 2019 (**Panel B**). Age-standardised DALY rate in 1990 (**Panel C**) and 2019 (**Panel D**). ASDR – disability-adjusted death rate, DALY – disability-adjusted life year

Further analysis of the disease burden across age groups revealed that the burden was concentrated in children under five years and adults over 80 years in both 1990 and 2019. In general, ASDRs and age-standardised DALY rates were higher in males than in females across all age groups. Notably, ASDRs peaked in the 95+ age group in both 1990 and 2019 (**Figure 4**, panels A–B), while age-standardised DALY rates were the highest in early childhood and remained low and stable throughout youth and middle age (**Figure 4**, panels C–D).

### Burden of disease attributable to unsafe WaSH by risk factor and cause

Figures S2–3 in the **Online Supplementary Document** present risk factor- and cause-specific ASDRs and age-standardised DALY rates from 1990 to 2019. Unsafe water source was the leading risk factor contributing to the highest number of deaths and DALYs throughout the study period (Figure S2 in the **Online Supplementary Document**), followed by unsafe sanitation and hygiene facilities. All three risk factors have exhibited declining trends over the 30 years examined.

Diarrhoeal diseases exhibited substantially higher ASDRs and age-standardised DALY rates compared to lower respiratory infections from 1990 to 2019. In 2019, the ASDR and age-standardised DALY rate for diarrhoeal diseases were 18.94 (95% CI = 13.59, 26.96) per 100 000 and 1040.75 (95% CI = 830.67, 1301.00) per 100 000, respectively (Table S3 in the **Online Supplementary Document**). These findings indicate that diarrhoeal diseases were the most heavily impacted by unsafe WaSH. Although both diarrhoeal diseases and lower respiratory infections showed decreasing trends in ASDRs and age-standardised DALY rates over the 30-year period, the overall rate of decline was more rapid for diarrhoeal diseases (Figure S3, panel A–B in the **Online Supplementary Document**).

## DISCUSSION

This study investigated the global trends in the burden of disease attributable to unsafe WaSH, covering 204 countries and territories over the past 30 years. We found that ASDRs and age-standardised DALY rates due to unsafe WaSH decreased globally, with the exception of an increasing ASDR in high-income North America. The greatest impact was concentrated in Africa and low SDI regions. Notably, U-shaped relationships were observed between the EAPCs of ASDRs, age-standardised DALY rates, and SDI. In contrast to WHO data, we found that the WaSH-related disease burden was higher in males than in females. The most vulnerable populations were children and the elderly. Unsafe water source and diarrhoeal diseases were identified as the leading risk factor and cause, respectively, highlighting the need for targeted interventions.

The global average disease burden due to unsafe WaSH has decreased by 64.51% from 1990 to 2019, likely due to improvements in quality of life and health systems. However, high-income North America was the only region that experienced an increase in ASDR during this period. Despite this increase, the ASDR in high-income North America remained relatively low compared to the global average in 2019 (**Table 1**, **Figure 2**). This finding underscores the region's overall favourable health outcomes but also highlights the need to address the factors contributing to the rising trend in WaSH-related ASDR to ensure continued progress in public health.

The observed negative correlation between ASDRs, age-standardised DALY rates, and SDI (**Figure 3**, panels A–B) may be attributed to better medical education, health care provider training, and social welfare in high SDI regions [18]. The U-shaped relationships between the EAPCs of ASDRs, age-standardised DALY rates, and SDI suggest that the impact of unsafe WaSH on disease burden is minimal in highly developed countries. However, the upward trend in EAPCs among high SDI regions indicates that continued attention to unsafe WaSH is necessary to maintain progress in these areas.

Interestingly, our study highlights some under-explored areas in the field of WaSH research. Due to limitations in the GBD database, we were only able to analyse data on diarrhoeal diseases and lower respiratory infections. To date, most evaluations of WaSH interventions have focused primarily on direct health hazards, with limited attention given to indirect and non-health impacts, such as mental health outcomes [19]. These underlying factors may affect a broader population than the pathological diseases mentioned above. Furthermore, in addition to children under five years, we identified individuals aged over 80 years as a high-risk group for diseases related to inadequate WaSH – an age group that has received limited attention in previous studies. This finding emphasises the need for further research to understand the specific challenges and vulnerabilities faced by this population.

Several limitations should be considered when interpreting the estimates from the GBD 2019 study. First, the exposure assessment approach does not account for all possible routes of exposure, such as contaminated shellfish consumption. Many diseases, particularly those related to water resource management and agricultural practices involving pathogens, cannot be quantified currently. Second, the GBD 2019 study defined high-quality piped water as the theoretical minimum risk exposure level (TMREL) for drinking water, without considering other improved water sources such as boreholes, tube wells, protected dug wells, protected springs, rainwater, and packaged or delivered water. For sanitation, the TMREL did not include the number of households sharing improved facilities when limited to less than two. These limitations may lead to inaccurate estimations of health impacts due to reporting bias. Third, the GBD 2019 data are estimated using a Bayesian meta-regression tool (DisMod-MR 2.1) rather than directly observed data, which may differ from the actual disease burden. Finally, unlike the WHO's classification and analysis of WaSH data [20], we did not conduct further examinations and comparisons of the distribution of the three risk factors across countries, sexes, and age groups, or their correlations with specific diseases. This omission limits our ability to explore the causes of WaSH-related illnesses and identify vulnerable populations for each risk factor. Therefore, more detailed and systematic investigations are necessary to assess the global health impact of unsafe WaSH in the future. Despite these limitations, our study has several strengths. To the best of our knowledge, this is the first comprehensive overview of the global disease and health burden attributable to unsafe WaSH using data from the GBD 2019 study. In contrast to the WHO database, which relies solely on data from member states, the GBD database offers more comprehensive coverage, encompassing nearly all countries and regions worldwide and drawing from more diverse data sources. By extracting and analysing the latest GBD data, our research provides a comprehensive and systematic evaluation of the global disease burden caused by unsafe WaSH from 1990 to 2019. This analysis may offer valuable insights for formulating environmental policies and health protection strategies for vulnerable populations, enabling them to face major global challenges such as water quality management, urbanisation, and climate change in the future.

## CONCLUSIONS

This 30-year study has demonstrated that while ASDRs and age-standardised DALY rates attributable to unsafe WaSH have decreased globally, the disease burden remains disproportionately high in Africa and low SDI regions. Long-term strategies should be tailored to address the increased vulnerability of children under five years and adults over 80 years, as well as the specific needs of certain territories. Governments should prioritise continuous improvements in access to safe water and sanitation facilities, while also raising public awareness about water safety and promoting the prevention and treatment of diarrhoeal diseases. Ongoing efforts to address the global burden of disease attributable to unsafe WaSH are crucial for progress towards achieving Sustainable Development Goal 6 and ensuring the health and well-being of vulnerable populations worldwide.

## Additional material


Online Supplementary Document
