# Supplementary material for: The global disease burden attributable to unsafe water, sanitation, and handwashing with unqualified facilities from 1990 to 2019
Source: J Glob Health. 2024 Aug 23;14:04162. doi: 10.7189/jogh.14.04162 (PMC11342018; doi:10.7189/jogh.14.04162)
Supplement: Online Supplementary Document [file jogh-14-04162-s001.pdf]

Table S1. Number of deaths and its EAPC from 1990 to 2019 due to unsafe water, sanitation and hygiene in different regions.

Region	Deaths		
	1990 (thousand), No. (95% UI)	2019 (thousand), No. (95% UI)	EAPC (%), No. (95% UI)
Global	3222.41 (2543.09-3961.34)	1656.89 (1198.86-2312.69)	-0.49 (-0.59, -0.32)
Andean Latin America	14.63 (11.25-18.03)	4.20 (2.54-6.21)	-0.71 (-0.80, -0.61)
Australasia	0.08 (0.04-0.12)	0.17 (0.09-0.25)	1.11 (0.74, 1.81)
Caribbean	16.76 (13.43-20.29)	7.38 (4.90-10.39)	-0.56 (-0.68, -0.40)
Central Asia	17.29 (14.03-20.82)	2.30 (1.48-3.29)	-0.87 (-0.90, -0.83)
Central Europe	1.29 (0.88-1.72)	0.66 (0.40-0.95)	-0.49 (-0.59, -0.37)
Central Latin America	51.66 (44.36-58.89)	11.77 (8.44-15.11)	-0.77 (-0.82, -0.71)
Central Sub-Saharan Africa	102.31 (69.41-138.16)	70.37 (46.42-100.56)	-0.31 (-0.50, -0.08)
East Asia	143.82 (108.30-183.48)	11.93 (6.93-17.58)	-0.92 (-0.94, -0.89)
Eastern Europe	2.66 (1.96-3.41)	1.38 (0.72-2.12)	-0.48 (-0.65, -0.36)
Eastern Sub-Saharan Africa	383.02 (293.73-473.31)	220.39 (161.83-286.50)	-0.42 (-0.57, -0.23)
High-income Asia Pacific	2.11 (1.16-3.13)	3.52 (1.76-5.43)	0.67 (0.37, 0.91)
High-income North America	1.03 (0.51-1.60)	2.25 (1.50-3.05)	1.19 (0.66, 2.40)
North Africa and Middle East	126.67 (96.26-164.05)	33.85 (22.07-46.43)	-0.73 (-0.79, -0.66)

Oceania	3.73 (2.75-4.82)	4.43 (3.06-6.18)	0.19 (-0.11, 0.55)
South Asia	1448.25 (1059.17-1872.43)	749.93 (456.97-1185.11)	-0.48 (-0.63, -0.25)
Southeast Asia	255.94 (187.36-336.28)	90.93 (59.52-133.04)	-0.64 (-0.75, -0.51)
Southern Latin America	2.30 (1.71-2.88)	1.64 (0.91-2.37)	-0.29 (-0.46, -0.15)
Southern Sub-Saharan Africa	36.23 (28.03-46.46)	26.82 (18.66-38.04)	-0.26 (-0.39, -0.11)
Tropical Latin America	46.97 (38.48-57.57)	11.05 (7.50-14.63)	-0.76 (-0.83, -0.70)
Western Europe	1.73 (0.91-2.61)	2.49 (1.36-3.67)	0.43 (0.28, 0.66)
Western Sub-Saharan Africa	563.95 (406.01-721.73)	399.42 (297.91-512.87)	-0.29 (-0.49, -0.01)

EAPC, estimated annual percentage change; UI, uncertainty interval.

Table S2. ASDR and age-standardized DALY rate (per 100,000) due to unsafe water, sanitation and hygiene in different countries and territories, in 1990 and 2019.

Countries and territories	ASDR (per 100,000), No. (95% UI)		Age-standardized DALY rate (per 100,000), No. (95% UI)	
	1990	2019	1990	2019
Afghanistan	45.17 (23.65-70.45)	17.03 (10.36-24.13)	3225.04 (1500.07-5397.37)	1159.69 (777.50-1662.52)
Albania	3.93 (2.40-5.54)	0.39 (0.22-0.59)	428.79 (315.59-551.78)	144.86 (90.02-212.11)
Algeria	11.31 (6.54-18.03)	2.22 (1.33-3.22)	786.00 (437.22-1310.20)	178.74 (116.29-243.70)
American Samoa	14.51 (8.72-24.11)	6.42 (3.60-10.82)	436.17 (312.02-607.78)	290.79 (182.70-407.04)
Andorra	0.22 (0.11-0.37)	0.15 (0.07-0.25)	9.47 (5.76-14.35)	5.36 (3.30-8.02)
Angola	372.09 (192.62-589.39)	88.40 (59.71-130.82)	18302.72 (9317.81-29167.36)	3155.86 (2179.33-4448.74)
Antigua and Barbuda	7.83 (5.53-10.27)	4.30 (2.67-6.20)	338.97 (267.99-413.61)	245.57 (174.29-322.36)
Argentina	5.40 (3.94-6.81)	2.38 (1.27-3.50)	317.13 (258.59-374.93)	96.84 (60.04-133.59)
Armenia	9.98 (7.73-12.45)	0.91 (0.53-1.36)	923.45 (720.50-1149.18)	143.97 (100.55-196.67)
Australia	0.32 (0.17-0.49)	0.28 (0.16-0.42)	21.32 (13.13-31.86)	16.21 (8.98-26.99)
Austria	0.14 (0.06-0.23)	0.11 (0.07-0.16)	20.07 (11.73-31.44)	8.40 (5.32-12.64)
Azerbaijan	22.48 (16.56-29.15)	2.39 (1.29-3.81)	2017.88 (1506.10-2588.54)	295.11 (192.29-423.04)
Bahamas	6.01 (4.14-8.06)	2.83 (1.68-4.13)	310.97 (234.71-395.42)	196.94 (138.49-262.04)
Bahrain	5.40 (3.22-8.42)	2.46 (1.41-3.85)	202.35 (144.73-270.90)	151.34 (94.74-220.56)

Bangladesh	178.19 (122.43-244.01)	35.50 (18.09-70.63)	6515.36 (5074.79-8182.26)	1041.76 (664.16-1664.43)
Barbados	4.96 (3.46-6.55)	3.41 (1.84-5.04)	255.72 (196.23-318.09)	213.34 (144.03-287.85)
Belarus	1.11 (0.73-1.49)	0.21 (0.11-0.33)	183.60 (137.59-243.83)	83.35 (51.35-124.64)
Belgium	0.34 (0.19-0.51)	0.41 (0.24-0.60)	18.12 (10.93-28.03)	11.37 (7.00-16.98)
Belize	17.98 (14.37-21.88)	7.52 (5.05-10.36)	1030.95 (844.89-1230.92)	387.00 (296.10-483.99)
Benin	195.14 (143.75-263.13)	81.94 (51.54-125.07)	8802.75 (6553.84-11498.90)	3179.49 (1922.55-5024.95)
Bermuda	3.17 (2.05-4.36)	1.12 (0.60-1.69)	180.50 (134.94-233.06)	153.45 (100.16-215.11)
Bhutan	161.29 (89.81-254.09)	23.43 (11.04-43.01)	4393.37 (2383.63-6501.36)	728.56 (451.67-1084.53)
Bolivia (Plurinational State of)	55.44 (34.03-83.38)	12.29 (7.31-18.78)	3036.55 (1823.52-4583.59)	537.11 (364.88-751.06)
Bosnia and Herzegovina	0.88 (0.60-1.19)	0.20 (0.12-0.31)	228.26 (154.49-314.93)	85.78 (43.54-138.59)
Botswana	140.99 (89.71-220.66)	48.37 (29.52-75.42)	5374.86 (3806.56-7489.62)	2038.93 (1338.01-2977.72)
Brazil	34.21 (28.05-41.37)	5.24 (3.60-6.89)	2384.03 (1946.14-2948.24)	280.62 (212.23-352.63)
Brunei Darussalam	2.04 (1.15-3.24)	1.48 (0.78-2.29)	50.40 (31.28-71.72)	32.02 (17.87-47.76)
Bulgaria	1.08 (0.64-1.58)	0.32 (0.17-0.49)	164.26 (98.44-237.00)	75.51 (36.86-125.79)
Burkina Faso	266.51 (188.84-363.35)	114.67 (78.06-170.19)	13132.20 (9355.81-17469.39)	4778.95 (3280.03-6672.64)
Burundi	234.67 (142.06-360.26)	124.89 (78.50-179.86)	9532.72 (6330.98-13139.38)	5077.43 (2924.03-7630.46)
Cabo Verde	81.67 (60.67-105.06)	22.57 (14.24-32.11)	4721.29 (3536.39-5992.71)	738.32 (531.19-957.14)

Cambodia	123.07 (80.05-183.33)	25.33 (15.71-38.73)	5128.26 (3659.31-6860.16)	796.26 (552.88-1086.34)
Cameroon	196.12 (129.81-265.72)	88.86 (56.05-125.19)	10277.73 (6195.39-13900.54)	4481.98 (2641.56-6499.33)
Canada	0.28 (0.14-0.44)	0.31 (0.20-0.44)	23.17 (13.53-35.44)	14.22 (8.52-21.40)
Central African Republic	333.61 (197.70-507.78)	254.21 (155.71-380.43)	14772.43 (8867.25-22655.62)	10361.41 (6769.31-14343.01)
Chad	289.14 (203.83-387.49)	190.27 (136.81-255.24)	14250.08 (10286.12-18482.38)	9817.13 (6944.73-13158.07)
Chile	5.92 (4.13-7.70)	1.13 (0.71-1.60)	223.22 (172.60-279.67)	54.15 (32.85-81.92)
China	14.55 (10.70-18.79)	0.87 (0.53-1.27)	1029.25 (793.60-1289.56)	91.40 (64.39-121.78)
Colombia	14.18 (11.41-16.69)	2.16 (1.38-3.11)	971.44 (791.00-1157.25)	166.94 (112.26-226.24)
Comoros	172.01 (94.44-262.10)	69.58 (46.43-101.93)	7710.56 (3881.85-11156.19)	2689.49 (1836.80-3673.14)
Congo	188.70 (119.06-272.16)	73.44 (44.83-112.72)	8238.37 (4704.79-12215.55)	2625.05 (1534.19-3743.41)
Cook Islands	8.00 (4.61-12.88)	2.76 (1.51-4.40)	319.80 (218.63-435.81)	205.01 (123.77-290.27)
Costa Rica	6.57 (4.92-8.16)	2.21 (1.35-3.20)	354.96 (272.90-446.70)	114.58 (71.53-164.22)
Croatia	0.41 (0.26-0.57)	0.18 (0.10-0.29)	103.97 (57.88-158.83)	50.23 (23.34-86.92)
Cuba	7.39 (5.54-9.18)	3.34 (1.99-4.98)	350.52 (285.42-421.56)	217.19 (156.30-285.98)
Cyprus	0.68 (0.37-1.16)	0.23 (0.12-0.37)	30.55 (18.96-45.55)	9.39 (5.29-15.69)
Czechia	0.27 (0.14-0.40)	0.43 (0.22-0.70)	77.48 (37.24-128.87)	43.22 (17.60-76.85)
Côte d'Ivoire	144.18 (102.80-196.87)	65.10 (40.68-94.24)	6543.77 (4745.24-8554.90)	2578.77 (1604.71-3872.38)

Democratic People's Republic of Korea	5.24 (3.18-7.77)	1.54 (0.89-2.38)	436.55 (290.64-635.27)	172.11 (110.29-251.93)
Democratic Republic of the Congo	147.77 (108.68-202.23)	80.96 (50.68-124.43)	6197.91 (4547.16-7985.69)	3033.36 (1839.47-4640.88)
Denmark	0.33 (0.18-0.48)	0.34 (0.20-0.49)	13.90 (8.55-21.35)	9.11 (5.61-13.67)
Djibouti	160.51 (104.87-229.68)	62.14 (37.93-92.26)	7742.81 (4796.43-10932.88)	2257.50 (1396.04-3243.92)
Dominica	10.10 (6.71-14.09)	5.05 (3.09-7.37)	440.12 (342.54-556.76)	294.35 (211.82-385.34)
Dominican Republic	37.28 (28.94-46.03)	8.80 (5.51-13.01)	2711.90 (2112.60-3379.74)	519.24 (360.23-737.68)
Ecuador	30.88 (23.13-39.49)	5.20 (2.83-8.02)	1794.68 (1426.19-2151.57)	253.71 (170.70-343.40)
Egypt	48.23 (38.08-60.15)	6.56 (3.97-9.76)	3851.20 (3014.67-4843.32)	551.84 (333.56-821.83)
El Salvador	45.11 (34.74-55.89)	7.46 (4.51-11.43)	2885.19 (2317.96-3454.34)	369.05 (261.32-493.73)
Equatorial Guinea	354.33 (165.75-610.12)	34.33 (20.57-54.36)	15048.51 (7270.31-25601.52)	998.03 (627.04-1524.35)
Eritrea	429.25 (161.04-689.81)	137.63 (80.98-253.25)	16274.23 (7821.98-24491.52)	5031.03 (3270.34-8547.13)
Estonia	0.56 (0.32-0.81)	0.22 (0.11-0.35)	133.49 (88.44-188.52)	82.69 (43.55-133.90)
Eswatini	128.98 (82.84-193.67)	77.93 (47.40-121.74)	5074.87 (3411.41-7265.73)	3214.79 (2094.97-4831.42)
Ethiopia	349.77 (194.64-521.00)	93.66 (61.24-129.56)	13656.00 (9177.46-18454.60)	3354.75 (2433.42-4418.66)
Fiji	42.28 (25.60-69.23)	15.86 (9.11-26.03)	1177.25 (835.50-1680.08)	608.42 (420.16-830.81)
Finland	0.52 (0.27-0.81)	0.07 (0.04-0.11)	17.29 (10.76-25.31)	4.85 (2.94-7.61)
France	0.47 (0.28-0.67)	0.20 (0.11-0.30)	20.74 (12.49-32.42)	8.04 (4.65-13.17)

Gabon	105.15 (67.11-154.11)	33.98 (19.78-54.76)	4171.89 (2604.12-6003.65)	1171.03 (711.12-1784.43)
Gambia	127.99 (88.61-180.08)	66.22 (43.03-97.78)	4978.09 (3428.84-6837.84)	2171.98 (1495.77-3095.31)
Georgia	5.01 (3.48-6.74)	0.75 (0.46-1.08)	494.48 (359.63-637.56)	158.30 (106.60-221.84)
Germany	0.20 (0.10-0.30)	0.20 (0.12-0.29)	10.60 (6.39-16.04)	7.25 (4.35-10.90)
Ghana	173.34 (113.26-241.68)	52.37 (34.51-75.40)	9550.23 (5619.49-13807.68)	1952.34 (1327.08-2732.35)
Greece	0.16 (0.07-0.26)	0.20 (0.08-0.33)	15.85 (10.23-23.54)	7.82 (4.94-11.25)
Greenland	1.87 (1.02-3.21)	0.67 (0.36-1.06)	76.15 (48.58-107.66)	29.37 (19.04-44.13)
Grenada	13.14 (8.21-18.20)	5.34 (2.95-7.84)	558.84 (407.99-710.34)	297.85 (216.11-387.70)
Guam	5.83 (3.44-9.86)	2.46 (1.33-4.02)	261.64 (176.96-352.60)	232.48 (137.44-335.46)
Guatemala	169.92 (145.44-192.03)	25.67 (18.67-33.95)	7727.86 (6762.93-8700.93)	1044.15 (776.33-1366.20)
Guinea	246.32 (165.99-343.90)	107.10 (69.55-157.85)	10825.45 (7813.26-14046.95)	4014.95 (2658.26-5640.61)
Guinea-Bissau	298.38 (190.97-445.32)	129.03 (83.88-193.02)	12347.23 (8462.16-17356.38)	4783.02 (3082.63-7097.09)
Guyana	39.50 (32.21-47.19)	16.43 (11.26-22.09)	2032.63 (1699.40-2401.30)	692.57 (517.27-904.57)
Haiti	154.31 (103.54-221.80)	49.07 (31.72-69.89)	9128.52 (6907.34-11547.78)	2814.83 (1768.30-4156.37)
Honduras	60.64 (45.17-83.25)	15.09 (8.53-27.86)	3602.97 (2856.29-4472.03)	678.64 (452.96-1002.70)
Hungary	0.23 (0.14-0.32)	0.49 (0.29-0.74)	153.25 (95.97-223.51)	76.35 (39.99-124.12)
Iceland	0.39 (0.18-0.62)	0.18 (0.09-0.29)	16.29 (9.64-24.73)	6.94 (4.25-10.15)

India	275.60 (180.60-374.91)	69.76 (41.28-111.64)	7803.99 (5766.67-10041.93)	1808.09 (1263.88-2585.86)
Indonesia	118.94 (74.67-178.05)	35.86 (21.06-54.16)	4350.82 (3435.50-5549.98)	1001.32 (708.56-1327.20)
Iran (Islamic Republic of)	9.44 (6.66-12.75)	1.65 (1.03-2.40)	618.49 (460.28-830.80)	169.10 (109.36-235.26)
Iraq	11.38 (5.49-18.90)	1.88 (1.18-2.77)	825.69 (373.58-1455.07)	201.20 (132.48-275.05)
Ireland	0.60 (0.26-0.95)	0.27 (0.13-0.43)	22.28 (14.39-31.62)	8.00 (5.11-11.83)
Israel	0.32 (0.19-0.47)	0.36 (0.22-0.51)	16.99 (10.61-26.00)	10.72 (6.87-15.93)
Italy	0.15 (0.07-0.23)	0.11 (0.07-0.17)	12.12 (7.41-19.25)	6.38 (3.69-10.31)
Jamaica	13.05 (10.64-15.71)	2.94 (1.86-4.25)	777.98 (641.63-937.14)	209.09 (155.09-271.21)
Japan	1.17 (0.57-1.79)	0.57 (0.29-0.88)	23.62 (13.93-34.41)	11.98 (7.07-17.66)
Jordan	4.62 (2.93-6.68)	1.31 (0.73-2.01)	278.73 (202.57-372.29)	119.71 (73.10-179.05)
Kazakhstan	10.98 (8.74-13.50)	1.11 (0.59-1.70)	972.46 (786.69-1188.91)	136.42 (96.36-186.86)
Kenya	133.57 (96.85-182.83)	82.17 (55.36-119.57)	5366.84 (4199.47-6738.80)	2898.23 (2116.64-3925.42)
Kiribati	224.80 (128.53-344.26)	86.19 (46.30-146.43)	5997.35 (4135.02-8346.57)	2111.70 (1350.07-3094.09)
Kuwait	1.72 (0.98-2.50)	1.44 (0.61-2.39)	124.03 (82.01-170.26)	99.64 (59.04-152.16)
Kyrgyzstan	12.96 (9.93-16.17)	1.73 (1.28-2.21)	1156.59 (905.38-1429.58)	235.85 (180.56-296.97)
Lao People's Democratic Republic	179.04 (95.89-279.93)	32.02 (20.45-48.83)	7600.42 (5163.95-10318.53)	1145.59 (715.04-1744.66)
Latvia	0.66 (0.43-0.93)	0.27 (0.14-0.41)	176.58 (119.28-248.71)	97.64 (54.35-148.01)

Lebanon	5.33 (3.46-7.59)	1.75 (0.97-2.84)	302.15 (225.86-405.02)	180.37 (114.71-250.58)
Lesotho	184.92 (128.21-257.43)	137.90 (88.74-210.44)	7877.20 (5993.85-10146.86)	5012.70 (3491.52-6933.47)
Liberia	235.48 (176.63-311.91)	87.36 (56.89-141.30)	13215.97 (9593.63-17547.10)	3673.48 (2252.85-5389.29)
Libya	8.01 (4.60-12.51)	1.89 (1.08-2.83)	494.50 (314.09-811.38)	186.85 (121.68-263.44)
Lithuania	0.66 (0.44-0.90)	0.35 (0.21-0.51)	163.69 (113.51-229.75)	101.31 (58.11-157.78)
Luxembourg	0.27 (0.15-0.41)	0.19 (0.11-0.30)	12.63 (7.89-19.30)	7.40 (4.42-11.38)
Madagascar	192.16 (149.14-251.35)	106.62 (69.37-158.48)	10471.00 (8498.46-12720.51)	4836.89 (3405.03-6560.63)
Malawi	249.95 (181.97-344.83)	92.73 (62.32-135.67)	12532.56 (9278.72-15916.36)	3284.27 (2305.79-4417.24)
Malaysia	10.26 (6.16-17.01)	5.68 (3.17-9.17)	326.90 (224.66-461.07)	183.17 (110.92-266.66)
Maldives	31.05 (21.18-45.92)	4.59 (2.97-6.80)	1398.97 (942.13-2051.51)	225.87 (170.20-286.02)
Mali	287.91 (200.89-387.34)	113.03 (63.04-189.42)	13353.95 (9419.47-17851.70)	4224.09 (2743.42-6564.60)
Malta	0.32 (0.15-0.52)	0.21 (0.10-0.36)	18.59 (11.69-27.56)	7.25 (4.39-10.87)
Marshall Islands	88.15 (47.21-146.78)	23.28 (11.96-41.43)	2250.81 (1422.02-3399.25)	716.69 (466.34-1048.80)
Mauritania	184.60 (124.69-255.35)	64.88 (39.05-97.13)	8430.44 (5346.71-11841.15)	2776.49 (1635.80-4176.75)
Mauritius	6.85 (5.38-8.36)	0.87 (0.50-1.28)	346.33 (266.86-431.54)	64.38 (34.79-99.68)
Mexico	31.21 (25.63-36.55)	3.87 (2.78-4.96)	1701.73 (1360.98-2040.71)	181.66 (128.33-234.07)
Micronesia (Federated States of)	79.13 (40.47-134.54)	18.63 (10.13-32.89)	2129.02 (1345.64-3204.10)	580.10 (385.48-831.12)

Monaco	0.21 (0.09-0.34)	0.20 (0.09-0.34)	8.90 (5.41-13.33)	5.79 (3.41-8.59)
Mongolia	32.32 (18.78-51.42)	2.73 (1.58-4.18)	2785.50 (1654.03-4416.44)	320.96 (231.65-430.38)
Montenegro	0.28 (0.17-0.41)	0.15 (0.08-0.22)	122.30 (73.16-180.62)	77.64 (41.35-119.52)
Morocco	42.49 (31.22-55.57)	5.91 (3.69-8.80)	3199.64 (2332.46-4274.72)	433.95 (287.28-636.86)
Mozambique	218.06 (125.58-329.06)	68.03 (39.22-102.87)	11774.26 (6347.05-19404.60)	2502.86 (1512.05-3658.55)
Myanmar	137.51 (70.20-222.25)	21.26 (14.21-31.29)	8069.68 (3575.85-13961.84)	851.38 (585.06-1182.55)
Namibia	135.76 (86.54-204.05)	53.14 (30.96-83.52)	4645.30 (2985.37-6696.09)	1968.56 (1209.32-2991.84)
Nauru	29.36 (17.42-48.63)	11.47 (6.52-19.39)	866.10 (590.79-1238.86)	466.90 (317.53-653.09)
Nepal	179.34 (102.66-267.88)	34.96 (19.75-60.09)	7050.13 (4992.80-9387.67)	1051.80 (732.21-1460.95)
Netherlands	0.23 (0.10-0.38)	0.22 (0.11-0.34)	9.04 (5.36-13.89)	5.88 (3.63-8.80)
New Zealand	0.76 (0.36-1.20)	0.40 (0.22-0.60)	36.48 (22.20-55.46)	25.58 (13.40-42.65)
Nicaragua	45.07 (36.52-54.29)	5.48 (3.57-7.96)	3417.97 (2768.68-4138.25)	331.39 (248.83-427.78)
Niger	398.10 (256.04-552.87)	144.12 (99.26-207.76)	22407.58 (15155.71-30771.91)	6906.54 (4651.81-9922.34)
Nigeria	301.04 (188.84-415.04)	109.91 (79.13-153.44)	16697.33 (11010.70-22808.36)	5663.49 (4364.08-7218.39)
Niue	18.23 (10.18-31.95)	5.95 (3.08-11.15)	571.31 (397.05-820.83)	317.44 (199.92-454.77)
North Macedonia	6.79 (4.37-9.41)	0.24 (0.14-0.38)	702.89 (452.75-959.43)	83.74 (45.34-134.90)
Northern Mariana Islands	8.03 (4.53-13.91)	5.28 (2.94-8.70)	290.95 (196.07-405.22)	291.10 (179.03-407.23)

Norway	0.48 (0.23-0.76)	0.30 (0.18-0.43)	13.42 (8.24-20.04)	6.97 (4.41-10.32)
Oman	17.78 (11.20-25.76)	3.86 (2.21-5.74)	637.91 (457.54-853.49)	205.43 (132.21-291.99)
Pakistan	160.48 (99.24-232.68)	64.26 (35.66-103.27)	5702.99 (4162.71-7309.72)	2188.05 (1545.24-3022.26)
Palau	25.77 (14.28-42.96)	10.87 (5.83-18.12)	755.64 (491.76-1094.40)	420.27 (275.58-586.71)
Palestine	8.87 (5.38-13.03)	2.78 (1.63-4.31)	388.83 (279.09-515.08)	182.73 (129.69-242.44)
Panama	9.99 (8.23-11.96)	4.57 (3.11-6.23)	689.00 (563.50-832.95)	320.84 (232.64-421.95)
Papua New Guinea	118.00 (83.36-168.52)	67.91 (44.36-106.56)	4100.15 (3050.26-5329.22)	2405.70 (1720.60-3266.76)
Paraguay	22.36 (16.31-30.30)	4.59 (2.74-6.86)	1192.62 (935.93-1470.11)	264.56 (187.48-358.27)
Peru	35.88 (25.55-47.11)	7.31 (4.17-11.13)	2175.39 (1604.17-2810.51)	371.53 (258.80-505.24)
Philippines	48.74 (34.47-68.03)	15.35 (9.90-22.52)	2363.63 (1741.54-2960.84)	568.98 (428.61-725.94)
Poland	0.51 (0.33-0.72)	0.29 (0.17-0.45)	107.56 (69.04-151.75)	44.78 (22.05-72.10)
Portugal	0.81 (0.52-1.13)	0.47 (0.27-0.71)	68.61 (47.95-92.06)	19.39 (12.22-28.93)
Puerto Rico	3.41 (1.67-5.26)	2.08 (1.32-3.00)	167.93 (113.06-222.96)	183.30 (121.93-252.50)
Qatar	2.85 (1.64-4.38)	1.43 (0.78-2.24)	169.34 (118.46-226.97)	92.66 (56.09-137.27)
Republic of Korea	2.03 (1.12-3.35)	0.61 (0.31-1.00)	60.39 (38.03-87.74)	15.36 (8.68-24.82)
Republic of Moldova	4.85 (3.31-6.48)	1.12 (0.59-1.73)	484.38 (366.31-615.58)	186.57 (133.46-253.52)
Romania	3.28 (2.30-4.36)	0.47 (0.28-0.68)	419.09 (321.93-523.56)	118.80 (73.40-176.25)

Russian Federation	1.59 (1.22-1.96)	0.51 (0.29-0.76)	237.26 (180.07-302.37)	124.74 (81.65-180.61)
Rwanda	261.69 (161.13-392.66)	64.60 (40.82-98.01)	12746.57 (8879.60-16992.01)	2395.95 (1595.90-3472.72)
Saint Kitts and Nevis	23.43 (18.56-28.46)	7.27 (4.97-9.67)	964.72 (808.35-1148.01)	361.88 (262.04-466.18)
Saint Lucia	12.19 (9.70-14.75)	3.76 (2.55-5.07)	556.22 (461.78-663.26)	279.63 (204.85-363.35)
Saint Vincent and the Grenadines	16.57 (13.06-20.09)	6.31 (4.35-8.51)	811.12 (651.03-991.99)	336.84 (257.60-427.00)
Samoa	16.90 (8.79-29.62)	6.65 (3.41-11.89)	529.53 (346.04-764.53)	296.50 (174.96-431.56)
San Marino	0.15 (0.07-0.24)	0.10 (0.05-0.18)	8.62 (5.22-13.48)	4.80 (2.94-7.38)
Sao Tome and Principe	107.24 (79.58-138.61)	28.58 (16.98-41.71)	5824.66 (4332.48-7570.95)	938.89 (641.26-1276.57)
Saudi Arabia	25.63 (16.41-36.57)	2.83 (1.61-4.27)	1043.82 (721.73-1484.86)	154.18 (94.65-225.66)
Senegal	212.70 (152.78-288.01)	74.15 (48.63-108.37)	9929.13 (7491.99-12597.17)	2949.97 (1926.94-4091.55)
Serbia	0.50 (0.29-0.76)	0.24 (0.12-0.39)	130.35 (80.63-191.43)	64.79 (31.18-106.00)
Seychelles	12.33 (7.56-19.03)	7.42 (4.56-11.32)	356.09 (255.28-478.67)	248.31 (175.60-330.43)
Sierra Leone	248.91 (178.64-334.56)	105.21 (61.58-162.84)	12399.57 (8871.44-16358.44)	3781.04 (2063.13-5888.91)
Singapore	2.71 (1.47-4.00)	1.06 (0.46-1.70)	64.42 (41.10-90.26)	20.87 (11.39-30.93)
Slovakia	0.76 (0.40-1.17)	0.25 (0.13-0.41)	126.68 (72.83-192.83)	48.83 (22.64-88.81)
Slovenia	0.32 (0.17-0.49)	0.14 (0.07-0.22)	72.59 (40.58-116.02)	33.19 (15.97-58.82)
Solomon Islands	241.41 (143.79-359.46)	95.16 (57.76-153.05)	6339.28 (4214.27-9094.60)	2496.65 (1732.45-3672.42)

Somalia	285.26 (151.47-455.97)	157.98 (91.94-248.87)	11283.70 (7057.77-16142.91)	6069.59 (3957.47-8734.85)
South Africa	80.04 (59.13-108.75)	37.69 (25.48-56.96)	4305.71 (3346.66-5523.92)	1466.87 (1098.90-1965.07)
South Sudan	188.17 (125.29-271.22)	134.20 (88.37-193.00)	8005.34 (5505.78-11390.96)	5193.81 (3574.40-7222.07)
Spain	0.28 (0.15-0.42)	0.18 (0.10-0.27)	15.05 (9.53-22.74)	7.39 (4.36-11.83)
Sri Lanka	37.04 (22.78-53.26)	5.47 (3.14-9.17)	942.57 (677.65-1240.84)	202.21 (143.02-280.21)
Sudan	76.28 (34.38-136.26)	21.81 (12.63-33.28)	5554.30 (2221.00-10534.65)	1350.62 (800.34-2140.72)
Suriname	28.24 (21.39-36.51)	9.14 (5.98-13.43)	1596.41 (1268.48-1930.71)	490.12 (354.04-650.62)
Sweden	0.30 (0.14-0.49)	0.25 (0.16-0.37)	13.59 (8.16-21.02)	7.53 (4.87-11.43)
Switzerland	0.29 (0.14-0.45)	0.13 (0.07-0.20)	9.75 (6.11-14.56)	4.91 (3.13-7.37)
Syrian Arab Republic	7.89 (5.23-11.03)	1.96 (1.17-2.94)	597.80 (405.23-850.62)	174.49 (116.20-239.01)
Taiwan (Province of China)	1.98 (1.26-2.73)	0.71 (0.34-1.15)	112.41 (81.11-146.20)	47.93 (26.38-75.64)
Tajikistan	48.36 (38.23-59.55)	10.04 (6.54-14.85)	4058.82 (3221.32-4976.84)	799.61 (555.20-1134.57)
Thailand	23.81 (15.05-37.90)	8.10 (4.74-12.63)	836.31 (590.55-1199.89)	273.18 (193.97-366.33)
Timor-Leste	110.34 (63.28-170.20)	31.87 (18.02-53.59)	4561.79 (3022.78-6375.53)	1037.50 (578.01-1527.94)
Togo	211.86 (146.67-280.73)	106.62 (73.02-148.15)	11348.61 (7201.82-15041.28)	5199.85 (3095.35-7253.40)
Tokelau	37.82 (21.34-65.01)	8.57 (4.63-15.40)	1026.80 (672.07-1586.23)	345.17 (234.09-491.51)
Tonga	12.78 (7.95-20.66)	4.33 (2.44-7.02)	461.93 (340.20-614.24)	268.22 (189.78-362.18)

Trinidad and Tobago	9.05 (6.64-11.74)	2.48 (1.61-3.59)	389.66 (305.82-475.97)	182.19 (130.56-242.50)
Tunisia	6.00 (3.76-8.94)	1.86 (1.06-2.91)	375.08 (249.78-577.52)	171.95 (117.54-233.36)
Turkey	10.17 (6.35-14.74)	1.87 (1.14-2.74)	728.18 (488.26-1034.69)	193.47 (131.43-266.77)
Turkmenistan	29.24 (22.94-36.03)	2.38 (1.48-3.46)	2504.47 (1975.23-3071.86)	267.69 (189.49-361.01)
Tuvalu	65.14 (34.49-113.99)	14.67 (7.80-26.05)	1846.96 (1213.67-2730.33)	500.02 (329.30-712.19)
Uganda	144.39 (97.19-200.11)	65.64 (42.14-99.04)	6538.74 (4236.38-9173.54)	2216.67 (1513.95-3113.21)
Ukraine	0.80 (0.53-1.09)	0.58 (0.30-0.93)	142.66 (104.30-191.71)	126.15 (85.54-177.91)
United Arab Emirates	9.80 (4.58-15.30)	3.54 (1.50-5.88)	260.74 (169.25-364.91)	142.10 (80.97-215.32)
United Kingdom	0.47 (0.19-0.77)	0.33 (0.15-0.52)	12.48 (7.09-19.68)	7.39 (4.40-11.15)
United Republic of Tanzania	187.15 (135.57-253.27)	71.01 (45.88-106.27)	8089.05 (6062.76-10389.06)	2293.34 (1548.93-3267.62)
United States of America	0.29 (0.14-0.45)	0.33 (0.22-0.45)	26.10 (16.38-39.46)	14.74 (9.43-21.49)
United States Virgin Islands	3.04 (1.88-4.49)	1.66 (0.96-2.47)	201.84 (148.67-263.37)	170.19 (111.42-236.12)
Uruguay	3.60 (2.68-4.47)	1.65 (1.01-2.41)	192.61 (141.92-240.86)	65.08 (37.75-98.53)
Uzbekistan	15.98 (12.18-19.93)	2.27 (1.15-3.57)	1396.23 (1086.82-1718.98)	235.43 (159.89-322.78)
Vanuatu	97.82 (57.75-158.03)	51.78 (28.59-87.37)	2621.17 (1691.93-3898.69)	1468.71 (969.81-2073.17)
Venezuela (Bolivarian Republic of)	18.42 (15.13-21.42)	4.82 (3.15-6.71)	1156.95 (953.21-1341.28)	278.00 (191.88-372.74)
Viet Nam	19.16 (12.50-27.81)	4.38 (2.53-6.72)	921.39 (607.49-1297.70)	191.66 (137.84-252.52)

Yemen	87.02 (44.25-140.61)	22.39 (12.62-35.54)	6343.80 (2946.76-10696.82)	1574.28 (837.88-2662.14)
Zambia	205.69 (139.92-294.27)	77.78 (53.34-113.73)	9383.41 (6753.86-12558.88)	3015.29 (2203.05-4144.80)
Zimbabwe	80.90 (53.07-107.98)	83.92 (52.61-118.61)	2944.00 (2091.55-3766.53)	2909.64 (1920.41-3945.65)

ASDR, disability-adjusted death rate; DALY, disability-adjusted life year; UI, uncertainty interval.

Table S3. ASDR and age-standardized DALY rate (per 100,000) of diarrheal diseases and lower respiratory infections due to unsafe water, sanitation and hygiene from 1990 to 2019**.**

Year	ASDR, per 100,000, No. (95% UI)		Age-standardized DALY rate, per 100,000, No. (95% UI)	
	Diarrheal diseases	Lower respiratory infections	Diarrheal diseases	Lower respiratory infections
1990	55.49 (41.19-72.04)	8.44 (3.63-12.97)	3065.08 (2432.05-3677.58)	548.89 (238.06-854.53)
1991	55.08 (40.29-71.55)	8.28 (3.61-12.72)	3037.40 (2420.84-3661.44)	537.69 (236.72-840.74)
1992	54.14 (39.42-70.92)	8.19 (3.54-12.67)	2964.56 (2343.00-3600.56)	529.73 (231.19-827.84)
1993	52.19 (38.50-68.28)	8.01 (3.47-12.37)	2874.50 (2295.41-3472.01)	516.63 (223.95-809.52)
1994	50.47 (37.60-65.80)	7.86 (3.40-12.15)	2807.14 (2249.92-3390.64)	505.06 (218.87-790.27)
1995	48.78 (36.25-64.31)	7.71 (3.34-11.87)	2721.86 (2163.62-3296.26)	494.42 (214.01-770.09)
1996	47.25 (35.01-61.56)	7.52 (3.27-11.62)	2645.52 (2106.43-3213.12)	479.84 (209.02-752.69)
1997	46.19 (34.58-60.12)	7.39 (3.24-11.31)	2581.41 (2065.77-3107.90)	467.96 (204.32-732.43)
1998	45.13 (33.92-59.38)	7.22 (3.17-11.16)	2522.20 (2032.13-3038.18)	455.34 (201.85-710.10)
1999	43.83 (32.81-57.56)	7.05 (3.10-10.80)	2450.95 (1973.62-2958.64)	443.58 (197.42-685.64)
2000	42.33 (31.74-56.03)	6.88 (3.05-10.55)	2367.67 (1916.22-2864.48)	430.91 (192.13-666.88)
2001	40.71 (30.65-54.08)	6.66 (2.94-10.16)	2280.48 (1851.90-2748.77)	414.92 (185.84-638.19)
2002	39.04 (29.62-51.58)	6.49 (2.86-9.86)	2190.75 (1788.94-2635.94)	401.51 (178.88-615.06)

2003	37.29 (28.75-49.17)	6.31 (2.78-9.55)	2099.18 (1727.61-2524.65)	388.26 (174.44-594.58)
2004	35.41 (27.21-46.82)	6.11 (2.70-9.23)	2008.83 (1668.25-2407.23)	375.07 (169.33-574.68)
2005	34.18 (26.23-45.42)	5.95 (2.64-8.99)	1938.73 (1618.00-2318.05)	362.83 (163.41-550.39)
2006	33.35 (25.69-44.36)	5.80 (2.59-8.76)	1887.65 (1578.61-2257.77)	351.56 (160.46-534.52)
2007	32.25 (24.67-42.91)	5.63 (2.50-8.54)	1817.47 (1512.61-2174.45)	338.87 (152.68-512.27)
2008	31.21 (23.96-41.80)	5.46 (2.45-8.39)	1749.03 (1458.54-2094.78)	326.00 (148.88-498.28)
2009	29.61 (22.53-39.82)	5.25 (2.37-8.05)	1672.50 (1399.11-1992.69)	312.47 (143.39-479.59)
2010	28.37 (21.66-38.05)	5.06 (2.27-7.77)	1600.43 (1346.53-1885.05)	299.29 (134.66-458.55)
2011	27.50 (20.85-36.92)	4.87 (2.19-7.51)	1537.18 (1289.18-1822.23)	285.68 (128.90-439.49)
2012	26.00 (19.69-35.39)	4.71 (2.11-7.34)	1450.82 (1218.77-1731.80)	274.50 (124.59-424.49)
2013	24.89 (18.67-34.02)	4.61 (2.06-7.16)	1378.65 (1150.30-1654.79)	265.88 (118.89-408.84)
2014	23.59 (17.50-32.50)	4.48 (1.99-6.89)	1305.35 (1079.97-1575.35)	256.92 (114.52-395.66)
2015	22.46 (16.56-31.22)	4.33 (1.91-6.65)	1239.36 (1010.88-1517.84)	246.40 (109.37-377.95)
2016	21.46 (15.66-29.86)	4.16 (1.83-6.42)	1185.14 (958.54-1457.82)	234.01 (104.37-361.98)
2017	20.98 (15.33-29.65)	4.00 (1.75-6.21)	1159.29 (931.89-1431.21)	221.93 (99.11-344.10)
2018	19.90 (14.39-28.14)	3.87 (1.69-6.00)	1093.57 (868.29-1364.92)	212.51 (94.69-331.75)
2019	18.94 (13.59-26.96)	3.74 (1.66-5.83)	1040.75 (830.67-1301.00)	203.55 (90.59-321.55)

ASDR, disability-adjusted death rate; DALY, disability-adjusted life year; UI, uncertainty interval.

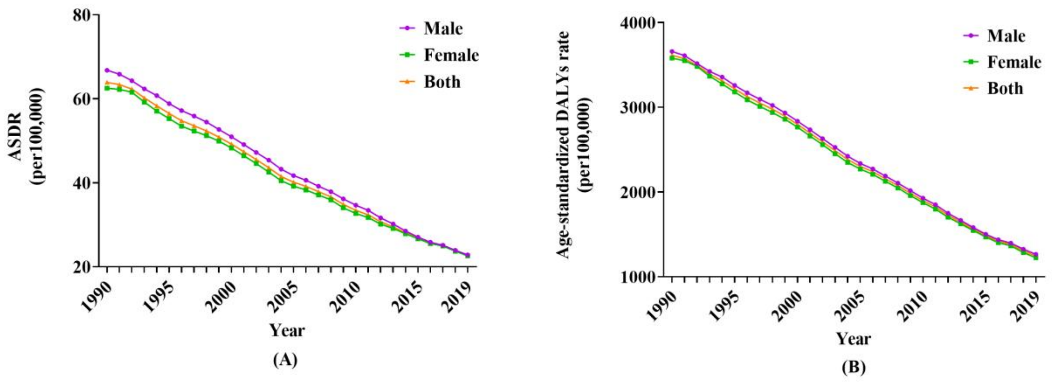

Figure S1. ASDR (A) and age-standardized DALY (B) rate of both sexes due to unsafe water, sanitation, and hygiene from 1990 to 2019. ASDR, disability-adjusted death rate; DALY, disability-adjusted life year.

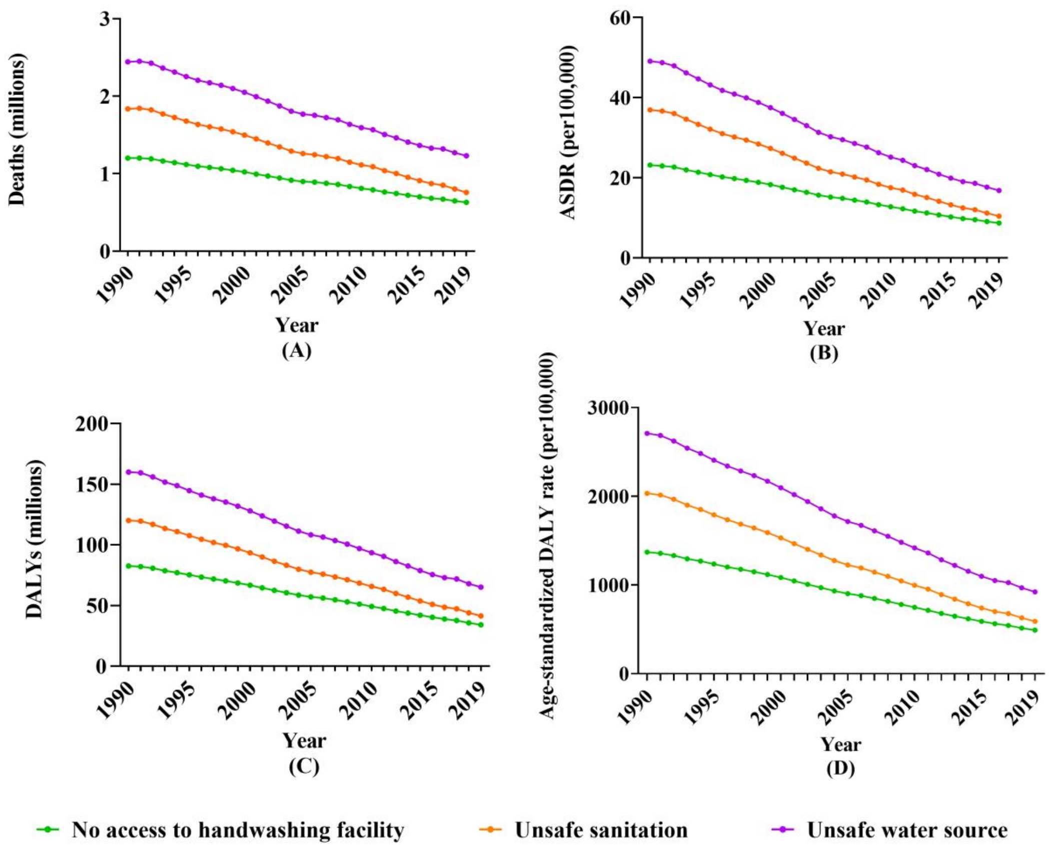

Figure S2. Disease burden caused by three risk factors of unsafe water, sanitation, and hygiene from 1990 to 2019. Number of deaths (A) and ASDR (B) from 1990 to 2019. Number of DALYs (C) and age-standardized DALY rate (D) from 1990 to 2019. *ASDR, disability-adjusted death rate; DALY, disability-adjusted life year.*

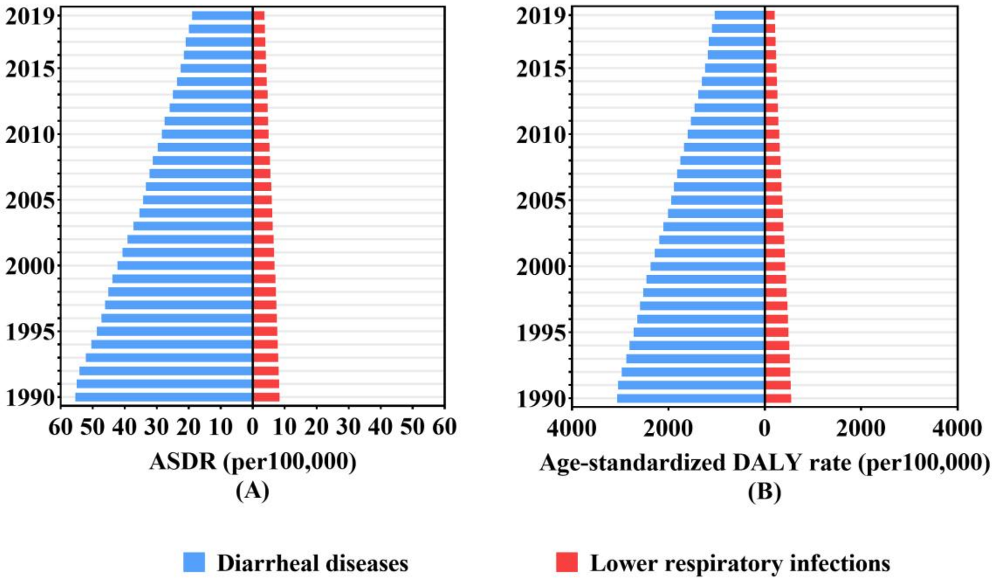

Figure S3. Comparison of ASDR (A) and age-standardized DALY rate (B) of different diseases caused by unsafe water, sanitation, and hygiene from 1990 to 2019 globally. Blue bar indicates diarrheal, red bar indicates lower respiratory infections. *ASDR, disability-adjusted death rate; DALY, disability-adjusted life year.*
